# Sex Differences in the Joint Trajectories of Depressive Symptoms and Body Mass Index From Adolescence to Early Adulthood: Longitudinal Observational Study

**DOI:** 10.2196/72722

**Published:** 2025-09-10

**Authors:** Jing Chen, Rui Shan, Wen Yuan, Qiong Wu, Yang Yang, Yi-Hang Yang, Jing-Yao Liu, Wu-Cai Xiao, Shanghang Zhang, Li-Ming Wen, Xiao-Rui Zhang, Zheng Liu, Yi Song

**Affiliations:** 1 Department of Maternal and Child Health, School of Public Health, Peking University, 38 Xueyuan Road, Haidian District, Beijing, 100191, China, 86 01082801222 ext 109; 2 Institute of Child and Adolescent Health, School of Public Health, Peking University, Beijing, China; 3 Institute of Social Science Survey, Peking University, Beijing, China; 4 The School of Computer Science, Peking University, Beijing, China; 5 School of Public Health, The Faculty of Medicine and Health, University of Sydney, Sydney, Australia; 6 Department of Pediatrics, Peking University People's Hospital, Beijing, China

**Keywords:** heterogeneity, trajectory, depressive symptoms, body mass index, sex

## Abstract

**Background:**

Adolescence is a critical transitional period between childhood and adulthood, marked by dramatic changes in physical and psychosocial health. Adolescents are vulnerable to both depression and adiposity, but how these conditions evolve over time from adolescence to early adulthood and whether sex differences exist remains unclear.

**Objective:**

This study aims to first identify the population heterogeneity in the joint trajectories of depressive symptoms and BMI from adolescence to early adulthood and then explore the sex differences in the joint trajectories.

**Methods:**

In this study, we adopt the latent class trajectory modeling to identify the combined trajectories of depressive symptoms and BMI from adolescence at baseline to early adulthood at follow-ups using a longitudinal study (2010-2020). We used the multinomial logistic regressions to examine the sex-specific associations with the trajectory classifications.

**Results:**

Our results found that individuals’ depressive symptoms and BMI might not always change parallelly from adolescence to early adulthood. Instead, some individuals appeared to be prone to depressive symptoms or elevated BMI, while others were multimorbid with both of them. Moreover, our study identified a clear sex-specific pattern in the joint trajectories of depressive symptoms and BMI: the females were at a higher risk of developing depressive symptoms but remained relatively stable weight status over time (odds ratio [OR] 0.68, 95% CI 0.52-0.89), while the males were at a lower risk of developing depressive symptoms but with an increasing risk of developing adiposity over time (OR 1.83, 95% CI 1.35-2.49).

**Conclusions:**

Depressive symptoms and BMI might not always change in parallel from adolescence to early adulthood, and there is a clear sex-specific pattern in the joint trajectories of depressive symptoms and BMI. This will inform the design of future sex-specific interventions that match the distinguished profiles in male and female participants during the period of adolescence and early adulthood, respectively, thus maximizing the intervention effects in preventing both depression and adiposity in early life.

## Introduction

Adolescence and early adulthood are critical periods transforming from childhood to adulthood with dramatic changes in physical and psychosocial health [1,2]. The health condition during adolescence and early adulthood not only lays a solid healthy foundation for the following life course but also sustains long-term health effects to the next life cycle (ie, intergenerational transmission). Indeed, many adult-prevalent, noncommunicable diseases, such as depression [3] and adiposity [4], start in adolescence or early adulthood. Globally, the prevalence of elevated depressive symptoms and obesity among adolescents has risen rapidly in recent decades [5,6] from 24% to 37% [7] and from 0.7% to 5.6% [8], respectively. Early adulthood is also crucial for both brain development and obesity prevention [9,10].

Multimorbidity refers to the coexistence of 2 or more long-term health conditions or diseases in an individual. Focusing on multimorbidity is critical, as targeting each health condition or disease in isolation can be ineffective and inefficient. To illustrate, if an intervention only focused on obesity by providing behavior change techniques to improve diet or physical activity behaviors, it would have neglected to provide emotional support for the vulnerable individuals, leaving them exposed to the potential adverse intervention effects such as body image dissatisfaction, low self-esteem, or even eating disorders [11]; by contrast, if an intervention targeted both depression and comorbid adiposity, it could combine elements of psychotherapy (eg, cognitive behavioral interventions) with healthy lifestyle enhancement, comprehensively covering both the physical and psychological health of intervention recipients [12].

To locate the time window when the multimorbidity interventions come in, it is important to clarify the longitudinal trends in depression and adiposity over time. For example, individuals with obesity at both age 13 and afterward had significantly higher risks of developing diabetes mellitus in adulthood, compared to those with obesity only at age 13 [13]. Another study pointed out that the trajectory of depressive symptoms over 10 years, rather than the one time point, had important health effects [14]. However, most existing studies typically assessed depression and adiposity cross-sectionally at one time point [15,16] but failed to capture their changes over time.

Researchers have called for breaking through the bottleneck of the “one-size-fits-all” intervention approach. One way to develop targeted interventions is to explore the sex-specific patterns of the coexistence of depression and adiposity [17]. Indeed, epidemiological studies have shown a higher risk of depression in females than males [18-21], while a higher risk of adiposity in males than females [22,23]. These findings might be attributable to the sex differences in individual- and family-level factors [17,24-27].

It is important to uncover the population heterogeneity in the longitudinal trends of depression and adiposity over time and to further explore whether the sex differences exist. Findings from such research could equip us to develop personalized, sex-specific interventions that consider the individual differences in the risk profile over time: depression alone, adiposity alone, or depression and adiposity simultaneously. For these reasons, we aimed to first identify the population heterogeneity in the joint trajectories of depressive symptoms and BMI from adolescence to early adulthood and then explore the sex differences in the joint trajectories.

## Methods

### Data Source and Study Population

The data source of this study was the China Family Panel Studies (CFPS), a nationally representative survey conducted by Peking University [28]. CFPS was first launched in 2010 with 5 subsequent rounds of surveys conducted in 2012, 2014, 2016, 2018, and 2020. The target sample of CFPS represents 95% of the population in China. Specifically, CFPS used a multistage probability-proportional-to-size sampling method and randomly sampled 640 communities nested within 144 counties from 24 provinces and 32 townships in Shanghai. Survey participants included all economically connected household members. CFPS has collected rich data at the individual, family, and community levels [28]. In this study, we included adolescents aged 10‐19 years without overweight 25 or depressive symptoms 26 at baseline, while some of them would enter the period of early adulthood during the 10-year follow-up surveys. To be eligible, participants should also have at least 2 measurements of BMI, BMI (kg/m^2^)=weight (kg)/(height [m])^2^ and at least 2 measurements of depressive symptoms available between 2010 and 2020.

### Sex

The exposure variable of this study was the sex of the adolescents, referring to the differences in biological aspects between males and females.

### Outcome Variables

#### Depressive Symptoms

Depressive symptoms were measured using the 20- or 8-item Center for Epidemiologic Studies Depression Scale (CES-D) during the 4 rounds (2012, 2016, 2018, and 2020) of surveys from 2012 to 2020. The CES-D has been validated and widely used to screen for depressive symptoms globally and in China [29-32]. To ensure comparability of outcomes measured by the 20- or 8-item CES-D, we converted the outcomes derived from the 8-item CES-D to the corresponding values measured by the 20-item CES-D based on the equipercentile equating method. We also calculated the *z* score of depressive symptoms: (score–mean score)/the SD of the scores.

#### BMI

Participants self-reported their height and weight in the CFPS questionnaires during the 6 rounds of surveys: 2010, 2012, 2014, 2016, 2018, and 2020. Studies have indicated that self-reporting data of height and weight are often highly correlated with the corresponding measurement data [33,34]. CFPS had very strict and standard criteria to control the quality of self-reporting data (Multimedia Appendix 1). Moreover, considering that the height and weight data of adolescents aged 10 to 15 were obtained from both the adolescents and their parents, we have comprehensively considered these data as follows: (1) when adolescents’ self-reported data and parents’ proxy-reported data were both available, we prioritized the adolescents’ self-reported responses as the primary data source. Meanwhile, we compared the data from those 2 sources and found that there was almost no difference. (2) For participants with only one available data source (either adolescents’ self-report or parents’ proxy report), the existing responses were retained for analysis. We then calculated the BMI *z* score to classify adolescents’ weight status relative to their age and sex based on the World Health Organization standard [35]. That is, a BMI *z* score higher than zero means that adolescents’ weight status was above the average weight status among those of the same age and sex. According to the World Health Organization standard [35], we defined adolescents with a BMI *z* score range of –2 to 1 as normal weight.

### Covariates

We identified covariates based on our literature review and domain knowledge for the present topic [18,36-38]. We grouped the covariates into 2 classifications: the individual- and family-level covariates. The individual-level covariates included age (old or young), area (urban or rural), region (western or central or eastern), high birth weight (yes or no), preterm birth (yes or no), and the only child (yes or no) at baseline. The family-level covariates include parents’ education level (college or above, or high school or below), whether the parents were obese or not, whether the parents were depressive or not, per capita household net income (high or low), left-behind children (yes or no), and primary caregiver (parents or other) at baseline.

### Statistical Analyses

First, we adopted the latent class trajectory modeling to identify the combined trajectories of depressive symptoms and BMI and classified the participants into heterogeneous groups [39,40]. To decide the suitable number of trajectories, we comprehensively considered multiple parameters such as the average of maximum probabilities, odds of correct classification, and so on (Tables S1 and S2 in Multimedia Appendix 2). We also validated the identified trajectories by analyzing their associations with subjective well-being and physical activity (Tables S3 and S4 in Multimedia Appendix 2).

Second, we used the multinomial logistic regression models to examine the association of sex with the participants’ classifications. We used 3 models to ensure the robustness of the study findings. In Model 1, we analyzed the crude association without covariates; in Model 2, we analyzed the association with the individual-level covariates only, including age, area, region, high birth weight, prematurity, and being the only child; in Model 3, we analyzed the association with both the individual- and family-level covariates, including age, area, region, high birth weight, prematurity, being the only child, father’s and mother’s education, obese status of father and mother, depression status of father and mother, per capita household net income, left-behind children, and primary caregiver. We also conducted sensitivity analyses by (1) using multiple imputations by chained equations [41] to account for the missing values of covariates; (2) using the Chinese criteria to define wasting [42], overweight, or obesity [43] in adolescents; (3) using the data from the 5 rounds of surveys: 2010, 2012, 2014, 2016, and 2018, excluding the data from 2020 (during the COVID-19 pandemic); (4) identifying the combined trajectories of depressive symptoms and BMI among males and females, respectively. All statistical analyses were performed with R (version 4.4.2; R Core Team). “LCTMtools” [39] and “mice” packages were used for trajectory analyses and multiple imputations, respectively. Statistical significance was determined by a 2-sided *P*<.05.

### Ethical Considerations

This cohort study was conducted based on the guidelines for reporting observational studies: the Strengthening the Reporting of Observational Studies in Epidemiology (STROBE) Statement [44]. This study was ethically approved by the Peking University Institutional Review Board (IRB00001052-22091, IRB00001052-14010). The data used for this study were deidentified. For each completed individual questionnaire, respondents were compensated—either in cash or as phone credit—at an average value of approximately US $6, with the exact amount varying by region and survey mode.

## Results

### Study population

**Figure 1.**
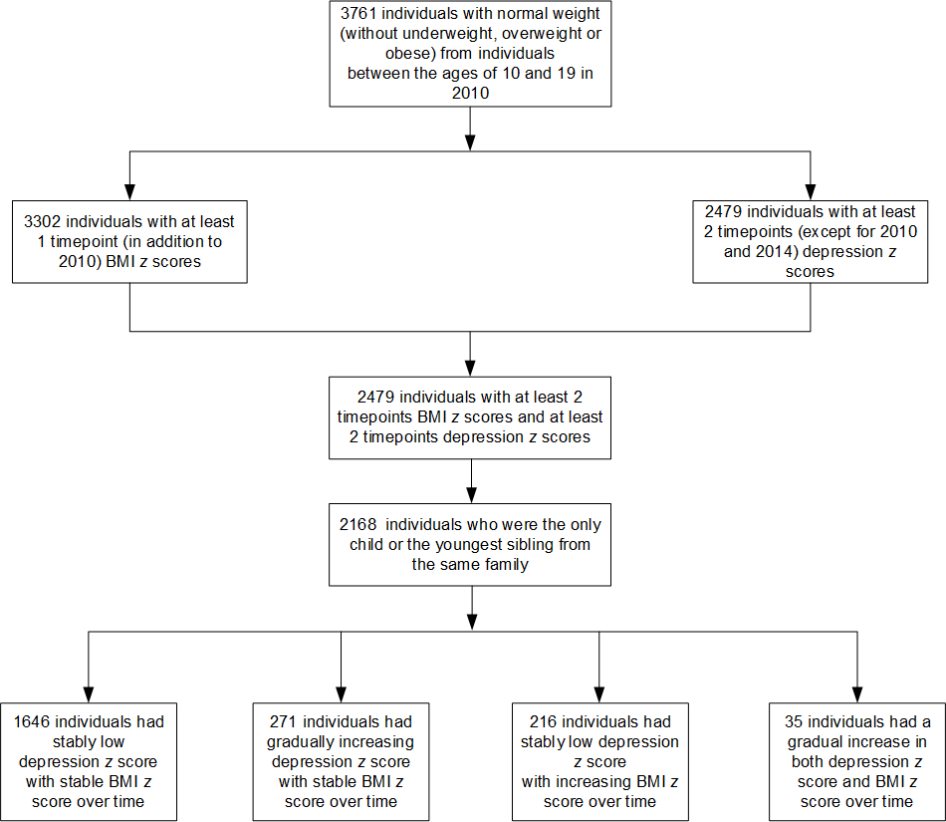
Selection of study population based on World Health Organization standards of BMI *z* score.

Figure 1 shows the flowchart of the selection process of the study population. Among 3761 adolescents (aged 10‐19 years) with normal weight in 2010, 2479 individuals had at least 2 time points of measurement of depression as well as 2 time points of measurement of BMI from 2010 to 2020. Finally, we included 2168 individuals who were the only child or the youngest sibling from the same family to meet the requirement of statistical modeling for independence between individuals. The characteristics between the included 2168 individuals and the excluded 1593 individuals were broadly comparable (Table S5 in Multimedia Appendix 2 ).

### Description of the Joint Trajectories of Depressive Symptoms and BMI

**Figure 2.**
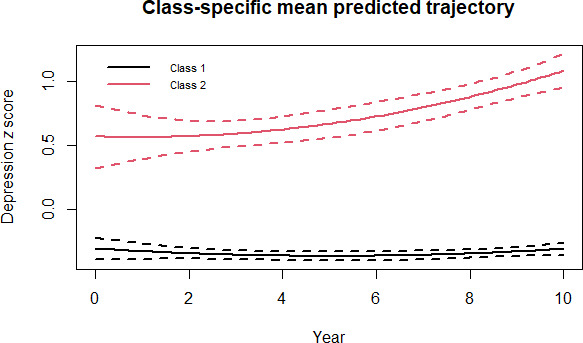
Classification of depression z score trajectory among individuals selected based on the World Health Organization standards Class 1: 1862 individuals; Class 2: 306 individuals.

Figures2  3 show the trajectories of depressive symptoms and BMI *z* score over time, respectively. The study participants were classified into 4 types of joint trajectories of depressive symptoms and BMI: (1) “health-sustaining group” represented by stably low depression *z* score with stable BMI *z* score over time (1646/2168 individuals, 75.92%); (2) “depression-dominant group” characterized by gradually increasing depression *z* score with stable BMI *z* score over time (271/2168 individuals, 12.50%); (3) “adiposity-dominant group” marked with stably low depression *z* score with increasing BMI *z* score over time (216/2168 individuals, 9.96%); (4) “multimorbidity group” with gradual increase in both depression *z* score and BMI *z* score over time (35/2168 individuals, 1.61%).

**Figure 3.**
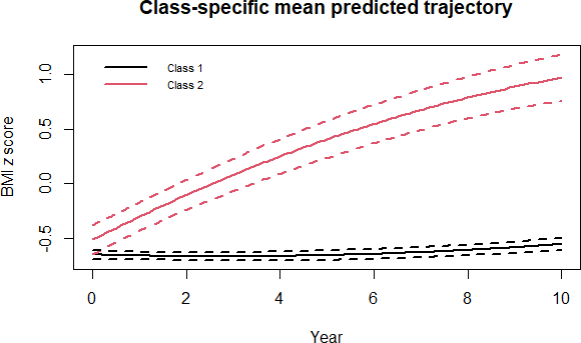
Classification of BMI z score trajectory among individuals selected based on the World Health Organization standards. Class 1: 1917 individuals; Class 2: 251 individuals.

We further validated the classifications of joint trajectories of depressive symptoms and BMI by analyzing their associations with subjective well-being scores and physical activity levels. Concerning the subjective well-being scores, individuals with stable depressive symptoms had higher scores than their counterparts. Concerning physical activity levels, individuals with stable depressive symptoms or stable BMI *z* score over time tended to exercise more often than the remaining individuals (Tables S3 and S4 in Multimedia Appendix 2).

### Association of Sex with the Joint Trajectories of Depressive Symptoms and BMI

**Figure 4.**
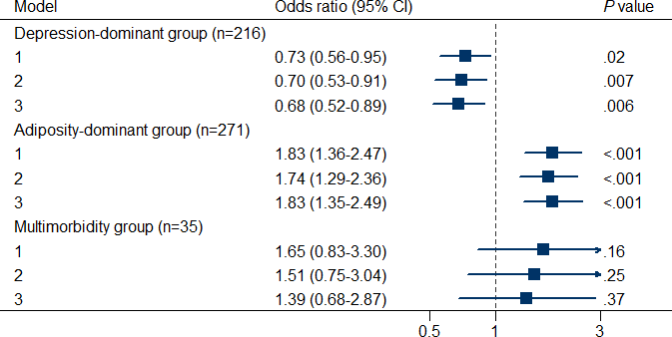
Sex differences in the joint trajectories of depression *z* score and BMI *z* score among individuals selected based on the World Health Organization standards with complete covariate data (before multiple imputations)

From adolescence to early adulthood, the females occupied the majority (n=168/306, 54.9%) in the trajectory of gradually increasing depressive symptoms (N=306) over time; in comparison, the males occupied the majority (n=163/251, 64.9%) in the trajectory of gradually increasing BMI (N=251) over time. There were significant sex differences in the 4 types of joint trajectories of depressive symptoms and BMI. Specifically, the proportion of males in the depression-dominant group (116/271, 42.80%) was lower (*P*=.02) than that in the health group (833/1646, 50.61%). The proportion of males in the adiposity-dominant group (141/216, 64.98%) was higher (*P*<.001) than that in the health group. No difference was found in the proportion of males in the multimorbidity group (22/35, 62.86%) compared with the health group (*P*=.21). As shown in Figure 4, the results from Model 1 to Model 3 consistently showed that the females were more likely to be classified into the depression-dominant group (OR 0.68, 95% CI 0.52-0.89); in comparison, the males were more likely to be classified into the adiposity-dominant group (OR 1.83, 95% CI 1.35-2.49).

### Sensitivity Analyses

The results from our sensitivity analyses were similar to those from the main analyses. Figure S1 in Multimedia Appendix 3 shows the results of using multiple imputations by chained equations. Figures S2-S5 in Multimedia Appendix 3 show the results of using the Chinese criteria to define wasting [42], overweight, or obesity [43] in adolescents. Figures S6-S8 in Multimedia Appendix 3 show the results of using the data of the 5 rounds of surveys: 2010, 2012, 2014, 2016, and 2018, excluding the data from 2020 (during the COVID-19 pandemic). Figures S9 and S10 in Multimedia Appendix 3 show the trajectories of depressive symptoms and BMI among males and female participants, respectively.

## Discussion

### Summary of Study Findings

This study used the population-based, nationally representative cohort study in China and showed that the joint trajectories of depressive symptoms and BMI from adolescence to early adulthood varied greatly and were classified into 4 groups with distinct characteristics: health-sustaining, depression-dominant, adiposity-dominant, and multimorbidity groups. Moreover, the joint trajectories showed a clear sex-specific pattern: females were at a higher risk of developing depressive symptoms but maintained a relatively stable weight status over time, while males were at a lower risk of developing depressive symptoms but showed an increasing risk of developing adiposity over time.

### Interpretation of Study Findings

Around a quarter of the participants were prone to depression or adiposity from before to during the COVID-19 pandemic, mirroring the health vulnerabilities during adolescence and early adulthood also shown in other studies [45,46]. Among these high-risk individuals, special attention should be given to those affected by both depression and adiposity, which could be interpreted by the shared genetic predisposition and brain circuitries that regulate both mood responses and homeostatic functions [47-49].

Interestingly, not all trajectories were parallel in the longitudinal changes in depressive symptoms and BMI. A possible interpretation is that the interaction between depression and BMI is a cumulative effect with some delays [50] rather than an instantaneous effect. This reminds us to continuously monitor and track the health status during adolescence and early adulthood with single depression or adiposity for a longer time and provide timely intervention to prevent the occurrence of multimorbidity.

Our findings, the female and male individuals showing a distinct pattern of the joint trajectories of depressive symptoms and BMI from adolescence to early adulthood, could be interpreted by the sex differences in genetic backgrounds and inflammation process [24,47,51]. Our proposed interpretations of the genetic backgrounds and inflammation process are one of the common causes of depression and adiposity. Studies [47] have suggested that some genetic loci, such as NEGR1 (neuronal growth regulator 1 gene, enables protein binding) or neural growth regulators, were associated with depression on the one hand and BMI and severe early-onset obesity on the other hand. It remained to further explore sex-specific genes or genetic pathways that determine both depression and adiposity. Concerning the inflammation process, another pathway potentially connecting depression with adiposity [24], females were shown to produce more inflammatory cytokines than males within a similar immune response [51].

Other potential interpretations of our findings were related to body dissatisfaction [25], stress coping [52,53], and personality traits [27,54]. Body dissatisfaction, being more prevalent in females than males [55], might result in poorer trajectories of depressive symptoms and eating disorders [25]. Regarding stress coping, studies suggested that females tend to be more sensitive to social stress than males [53], thus posing females at a higher risk of depression than males [52]. In addition, the social stress on body image is more specific to females, making them more likely to maintain a normal weight than males. Regarding personality traits, studies have indicated that low agreeableness, more prevalent in males [27], was associated with a greater increase in BMI [54]. However, it remains to explore the specific mechanisms of personality traits underlying our findings.

The biological mechanisms underlying our findings are likely multifaceted. Beyond previously discussed factors such as stress coping, which may concurrently contribute to both depression and adiposity, the bidirectional vicious cycle between depression and adiposity [56] could also play a significant role. For instance, depression might promote adiposity (and subsequent multimorbidity development), and adiposity might also promote depression, resulting in a self-reinforcing loop. The strength of these bidirectional relationships may differ between males and females, potentially explaining the observed sex differences in the joint trajectories from normal state to comorbid depression and adiposity. More specific mechanisms should be further elucidated through targeted investigations in the future.

### Comparison With Other Similar Studies

To the best of our knowledge, we have not found other studies that have explored the joint trajectories of depressive symptoms and BMI from adolescence to early adulthood. The existing studies have only focused on the trajectories of depressive symptoms or adiposity separately.

Concerning studies examining the trajectories of depressive symptoms, Kwong et al [20] found similar findings to ours that females were associated with the childhood-persistent trajectory of depressive symptoms compared with the stable-low trajectory. Zhang et al [57] also found a poorer trajectory of depressive symptoms in Chinese females than in males. Concerning studies examining the trajectories of adiposity, Koning et al [58] found that the proportion of males in the group with increasing BMI *z* score tended to be higher than that in the group with a gradually decreasing BMI *z* score, which was also consistent with our findings.

### Strengths and Limitations

Our study findings should be interpreted with caution. First, the height and weight data used for trajectory analyses were based on a self-report questionnaire. However, previous studies had found that self-reported height and weight data were often closely related to actual measured data [33,34], and the CFPS had strict criteria for controlling the quality of self-reported data (Multimedia Appendix 1), which could strengthen the reliability of our results. Second, a small proportion of study participants were classified into the multimorbidity group, limiting us to identifying potential sex differences compared to the reference group. Third, other potential confounding factors that could simultaneously influence depressive symptoms and BMI were not adjusted for due to unavailable data, such as individual dietary behaviors. Fourth, individuals’ health problems that could be the source of depression and adiposity were not clear. However, we have analyzed whether there were differences in the distribution of malignant tumors or endocrine diseases among different sexes based on available data and found no statistically significant results (*P*>.05). Moreover, the probability of developing other chronic diseases is low in adolescents or early adults aged 10 to 24. Therefore, individuals’ health problems will not have a significant impact on the results of our study.

Nevertheless, our study population was from a representative sample in China, which indicates great generalizability in the Chinese population. In addition, results from several sensitivity analyses were rather consistent, increasing the robustness of our study findings.

### Research Implications

Adolescence and early adulthood are a critical “watershed” period when multiple risk behaviors might emerge, and in the meanwhile, these risk behaviors can be prevented and healthy behavior habits can also be established. Thus, this transitional period is also a critical “window” period when prevention-oriented interventions might come in. Multimorbidity of depression and adiposity has been widely recognized, and relevant pilot interventions are currently in the design or early implementation stage [59]. Our study is unique in taking a sex-specific view of the combined longitudinal trends of depressive symptoms and BMI from adolescence to early adulthood. Our findings indicated that multimorbidity intervention for both depression and adiposity may benefit more from designing sex-specific intervention measures compared with the “one-size-fits-all” approach, as females tend to be depression-prone but adiposity-resistant while males show the opposite pattern.

### Conclusions

The findings of our study could pave the way for future multimorbidity interventions that aim to simultaneously prevent the 2 common health issues during adolescence and early adulthood: depression and adiposity. Data from the large, representative sample in China showed that the depressive symptoms and BMI might not increase or decrease simultaneously from adolescence to early adulthood over 10 years; instead, there emerged 4 distinct groups of trajectories in the changes of depressive symptoms and BMI: health-sustaining, depression-dominant, adiposity-dominant, or multimorbidity group. We also identified sex differences in the joint trajectories of depressive symptoms and BMI from adolescence to early adulthood. This will guide the development of future sex-specific interventions tailored to the distinct profiles of males and females from adolescence to early adulthood, respectively, thereby optimizing intervention effectiveness for preventing both depression and adiposity in early life.

## Supplementary material

10.2196/72722 Multimedia Appendix 1 Data quality control criteria for self-reported height and weight, data cleaning procedures, and definitions of well-being and physical activity measures.

10.2196/72722 Multimedia Appendix 2 Participant characteristics, model adequacy assessments for depression and BMI z score trajectories, and distributions of well-being and physical activity across trajectory groups, based on World Health Organization standards.

10.2196/72722 Multimedia Appendix 3 Results of sensitivity analyses.

## Abbreviations

CES-D: Center for Epidemiologic Studies Depression Scale
CFPS: China Family Panel Studies
OR: odds ratio
STROBE: Strengthening the Reporting of Observational Studies in Epidemiology
