# Correction: Sex Differences in the Joint Trajectories of Depressive Symptoms and Body Mass Index From Adolescence to Early Adulthood: Longitudinal Observational Study

**DOI:** 10.2196/84632

**Published:** 2025-09-30

**Authors:** Jing Chen, Rui Shan, Wen Yuan, Qiong Wu, Yang Yang, Yi-Hang Yang, Jing-Yao Liu, Wu-Cai Xiao, Shanghang Zhang, Li-Ming Wen, Xiao-Rui Zhang, Zheng Liu, Yi Song

**Affiliations:** 1 Department of Maternal and Child Health, School of Public Health, Peking University, 38 Xueyuan Road, Haidian District, Beijing, 100191, China, 86 01082801222 ext 109; 2 Institute of Child and Adolescent Health, School of Public Health, Peking University, Beijing, China; 3 Institute of Social Science Survey, Peking University, Beijing, China; 4 The School of Computer Science, Peking University, Beijing, China; 5 School of Public Health, The Faculty of Medicine and Health, University of Sydney, Sydney, Australia; 6 Department of Pediatrics, Peking University People’s Hospital, Beijing, China

In “Sex Differences in the Joint Trajectories of Depressive Symptoms and Body Mass Index From Adolescence to Early Adulthood: Longitudinal Observational Study” [1], the authors noted two affiliation errors.

The affiliation for author XRZ has been changed to:

Department of Pediatrics, Peking University People’s Hospital, Beijing, China

In addition, the affiliations for author YS have been amended so that the author is only affiliated with the following institution:

Institute of Child and Adolescent Health, School of Public Health, Peking University, Beijing, China

The correction will appear in the online version of the paper on the JMIR Publications website, together with the publication of this correction notice. Because this was made after submission to PubMed, PubMed Central, and other full-text repositories, the corrected article has also been resubmitted to those repositories.

## References

[1] Chen J, Shan R, Yuan W (2025). Sex differences in the joint trajectories of depressive symptoms and body mass index from adolescence to early adulthood: longitudinal observational study. JMIR Pediatr Parent.

