# Supplementary material for: Sex Differences in the Joint Trajectories of Depressive Symptoms and Body Mass Index From Adolescence to Early Adulthood: Longitudinal Observational Study
Source: JMIR Pediatr Parent. 2025 Sep 10;8:e72722. doi: 10.2196/72722 (PMC12422740; doi:10.2196/72722)
Supplement: Multimedia Appendix 1 [file pediatrics-v8-e72722-s001.docx]

**Text S1**

1. **The criteria of CFPS aim to control the quality of self-reporting height and weight**
2. **Steps for us to re-checked and cleaned the data of height and weight**
3. **The definitions of “Score of subjective well-being” and “Duration of physical activity per week”**

**The criteria of CFPS aim to control the quality of self-reporting height and weight**

| Year | Age range | Allowable height range  for input (cm) | Height range that  requires soft check (cm) | Allowable weight range  for input (kg) | Weight range that  requires soft check (kg) | Other |
| --- | --- | --- | --- | --- | --- | --- |
| 2010 | 0 to 1 | 48 to 100 | — | 0.5 to 30 | — | — |
| 2010 | 1 to 3 | 48 to 120 | — | 0.5 to 100 | — | — |
| 2010 | 3 to 6 | 48 to 160 | — | 0.5 to 100 | — | — |
| 2010 | 6 to 16 | 48 to 200 | — | 0.5 to 100 | — | — |
| 2010 | ≥ 16 | 50 to 240 | — | 15 to 300 | — | Soft check:  BMI ≤ 18.5 or BMI ≥ 27 |
| 2012 | < 16 | 50 to 200 | — | 0.5 to 100 | — | — |
| 2012 | ≥ 16 | 50 to 240 | < 100 or > 200 | 25 to 150 | < 40 or > 100 | — |
| 2014 | < 16 | 30 to 230 | — | 0.5 to 150 | — | — |
| 2014 | ≥ 16 | 50 to 240 | < 100 or > 200 | 25 to 150 | < 40 or > 100 | — |
| 2016 | < 16 | 30 to 230 | — | 0.5 to 150 | — | Soft check:  BMI < 18.5 or BMI > 27 |
| 2016 | ≥ 16 | 50 to 240 | < 100 or > 200 | 25 to 150 | < 40 or > 100 | — |
| 2018 | < 16 | 30 to 230 | > 200 | 0.5 to 150 | < 15 or > 100 | Soft check:  BMI < 18.5 or BMI > 27 |
| 2018 | ≥ 16 | 30 to 240 | < 100 or > 200 | 15 to 150 | < 40 or > 100 | Soft check:  BMI < 18.5 or BMI > 27 |
| 2020 | < 16 | 30 to 230 | > 200 | 0.5 to 150 | < 15 or > 100 | Soft check:  BMI < 18.5 or BMI > 27 |
| 2020 | ≥ 16 | 30 to 240 | < 100 or > 200 | 15 to 150 | < 40 or > 100 | Soft check:  BMI < 18.5 or BMI > 27 |

**Steps for us to re-checked and cleaned the data of height and weight**

1. Check the range of height and weight data, respectively.
2. Cleaned the data of height and weight less than 40 cm and 0.5 kg, respectively.
3. Cleaned the data of height and weight more than 240 cm and 150kg, respectively.
4. Calculate for BMI Z-score, and cleaned the BMI Z-score that was out of the range of -5 to 5.

**The definitions of “score subjective well-being”**

**and “the duration of physical activity per week”**

| Variable | Definition |
| --- | --- |
| Score of subjective well-being | Question: How much well-being do you feel?  Range of score: 0 to 10 (10 represents the highest score of subjective well-being) |
| Duration of physical activity per week | Question: How much hours (including physical education class) did you exercise last week? |
