# Supplementary material for: Sex Differences in the Joint Trajectories of Depressive Symptoms and Body Mass Index From Adolescence to Early Adulthood: Longitudinal Observational Study
Source: JMIR Pediatr Parent. 2025 Sep 10;8:e72722. doi: 10.2196/72722 (PMC12422740; doi:10.2196/72722)
Supplement: Multimedia Appendix 2 [file pediatrics-v8-e72722-s002.docx]

**Table S1 Model adequacy assessments of latent trajectory class models for depression Z-score (using Homoscedastic structure model A)**

| Number of classes | Number of parameters | Bayesian information criteria (BIC) | Proportion  per class % | average of maximum probabilities (APPA) | odds of correct classification (OCC) | Relative entropy values |
| --- | --- | --- | --- | --- | --- | --- |
| 1 | 4 | 16097.87 | / | / | / | / |
| 2 | 8 | 15400.12 | 86:14 | 0.948:0.838 | 3.539:26.515 | 0.764 |
| 3 | 12 | 15291.67 | 80:4:16 | 0.920:0.796:0.753 | 3.450:80.047:13.488 | 0.755 |
| 4 | 16 | 15248.56 | 77:16:4:3 | 0.898:0.697:0.704:0.780 | 3.345:11.520:29.949:99.764 | 0.731 |
| 5 | 20 | 15179.37 | 71:23:1:3:2 | 0.887:0.719:0.830:0.795:0.767 | 3.751:7.838:294.938:91.514:165.281 | 0.760 |

**Table S2 Model adequacy assessments of latent trajectory class models for BMI Z-score (using random intercept structure model C)**

| Number of classes | Number of parameters | Bayesian information criteria (BIC) | Proportion  per class % | average of maximum probabilities (APPA) | odds of correct classification (OCC) | Relative entropy values |
| --- | --- | --- | --- | --- | --- | --- |
| 1 | 5 | 24404.84 | / | / | / | / |
| 2 | 10 | 24035.50 | 88:12 | 0.925:0.801 | 2.324:21.239 | 0.678 |
| 3 | 15 | 23942.58 | 86:11:3 | 0.913:0.805:0.801 | 2.381:23.163:115.162 | 0.762 |
| 4 | 20 | 23941.58 | 65:3:16:16 | 0.750:0.790:0.644:0.769 | 2.411:97.336:6.153:15.014 | 0.544 |
| 5 | 25 | 23923.21 | 3:26:3:59:9 | 0.818:0.715:0.765:0.734:0.620 | 116.976:6.053:67.760:2.756:11.481 | 0.573 |

**Table S3 Distribution of subjective well-being score and physical activity level among two groups with different depression Z-score trajectories based on the WHO standards**

| Characteristics | Group E (1358) | Group F (227) |
| --- | --- | --- |
| Score of subjective well-being  (mean±sd) | 7.76±1.61 | 6.36±2.24* |
| Duration of physical activity per week  High (n, %) | 673 (49.56%) | 100 (44.05%) |

Group E indicates individuals with gradually stable depression Z-score over time;

Group F indicates individuals with gradually increasing depression Z-score over time.

Among 2168 individuals selected based on WHO standard, 1353 individuals had score of subjective well-being;

Among 2168 individuals selected based on WHO standard, 1585 individuals had duration of physical activity per week.

* Significant difference compared to the reference group (group E) according to the linear regression model.

**Table S4 Distribution of subjective well-being score and physical activity level among two groups with different BMI Z-score trajectories based on the WHO standards**

| Characteristics | Group G (1394) | Group H (191) |
| --- | --- | --- |
| Score of subjective well-being  (mean±sd) | 7.55±1.79 | 7.55±1.78 |
| Duration of physical activity per week  High (n, %) | 689 (49.43%) | 84 (43.98%) |

Group G indicates individuals with stable BMI Z-score;

Group H indicates individuals with gradually increasing BMI Z-score over time.

Among 2168 individuals selected based on WHO standard, 1353 individuals had score of subjective well-being;

Among 2168 individuals selected based on WHO standard, 1585 individuals had duration of physical activity per week.

**Table S5 Distribution of characteristics among included and excluded individuals based on the WHO standards**

| Characteristics,  n (%) | Class | Included individuals  (n = 2168) | Excluded individuals  (n = 1593) | *P* value of chisq-test among two groups |
| --- | --- | --- | --- | --- |
| Individual level | | | | |
| Sex  Male/Female | Male | 1112(51.29%) | 728(45.70%) | <.001 |
| Age  High/Low | High | 813(37.50%) | 806(50.60%) | <.001 |
| Area  Urban/Rural | Urban | 880(40.59%) | 660(41.43%) | .628 |
| Region  Western/Central/Eastern | Western | 694(32.01%) | 527(33.08%) | .386 |
|  | Central | 674(31.09%) | 462(29.00%) |  |
|  | Eastern | 800(36.90%) | 604(37.92%) |  |
| High birth weight  Yes/No | Yes | 66(3.82%) | 34(3.17%) | .430 |
| Premature  Yes/No | Yes | 870(64.54%) | 480(62.58%) | .393 |
| The only child  Yes/No | Yes | 1431(86.15%) | 1194(92.27%) | <.001 |
| Family level | | | | |
| Father's education  College or above/High school or below | College or above | 121(5.89%) | 78(5.39%) | .579 |
| Mother's education  College or above/High school or below | College or above | 82(3.99%) | 56(3.86%) | .911 |
| Obesity (father)  Yes/No | Yes | 546(33.33%) | 334(30.31%) | .105 |
| Obesity (mother)  Yes/No | Yes | 533(29.27%) | 307(25.06%) | .012 |
| Depression (father)  Yes/No | Yes | 475(23.97%) | 329(29.35%) | .002 |
| Depression (mother)  Yes/No | Yes | 664(33.38%) | 408(36.46%) | .090 |
| Per capita household net income  High/Low | High | 1064(52.00%) | 708(47.26%) | .006 |
| left-behind children  Yes/No | Yes | 141(8.68%) | 105(10.63%) | .113 |
| Primary caregiver  Parents/Other | Parents | 988(73.08%) | 537(68.32%) | .022 |
