# Supplementary material for: Sex Differences in the Joint Trajectories of Depressive Symptoms and Body Mass Index From Adolescence to Early Adulthood: Longitudinal Observational Study
Source: JMIR Pediatr Parent. 2025 Sep 10;8:e72722. doi: 10.2196/72722 (PMC12422740; doi:10.2196/72722)
Supplement: Multimedia Appendix 3 [file pediatrics-v8-e72722-s003.docx]

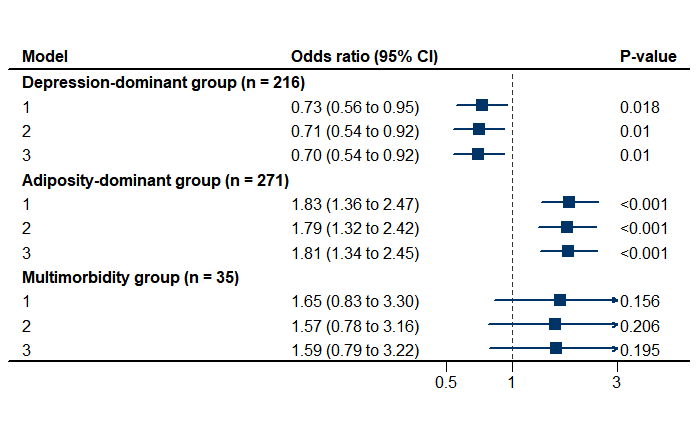


**Figure S1 Sex disparity in the joint trajectories of depression Z-score and BMI Z-score among individuals selected based on the WHO standards**

**with complete covariate data (after multiple imputations)**

Model 1: multinomial logistic regression model without covariate;

Model 2: multinomial logistic regression model adjusted for individual-level covariates, including age, area, region, high birth weight, premature, and the only child;

Model 3: multinomial logistic regression model adjusted for individual- and family-level covariates, including age, area, region, high birth weight, premature, and the only child, father’s and mother’s education, obese status of father and mother, depression status of father and mother, per capita household net income, left-behind children, and primary caregiver.

**
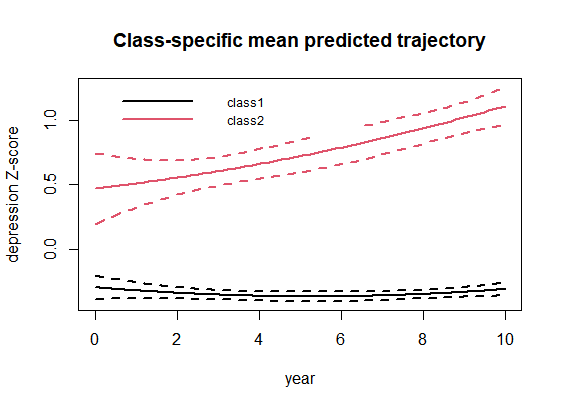
**

**Figure S2 Classification of depression Z-score trajectory**

**among individuals selected based on the Chinese standards**

Class1: 1657 individuals; Class 2: 246 individuals

**
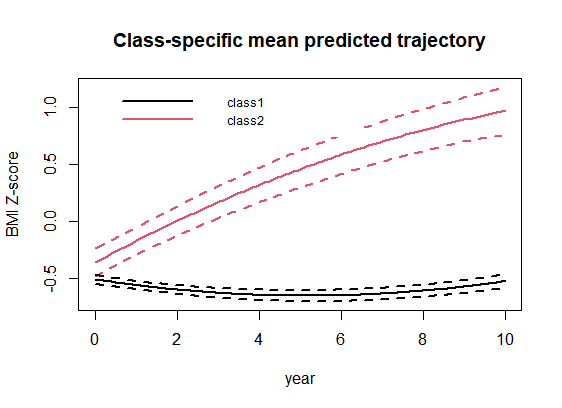
**

**Figure S3 Classification of BMI Z-score trajectory**

**among individuals selected based on the Chinese standards**

Class1: 1643 individuals; Class 2: 260 individuals


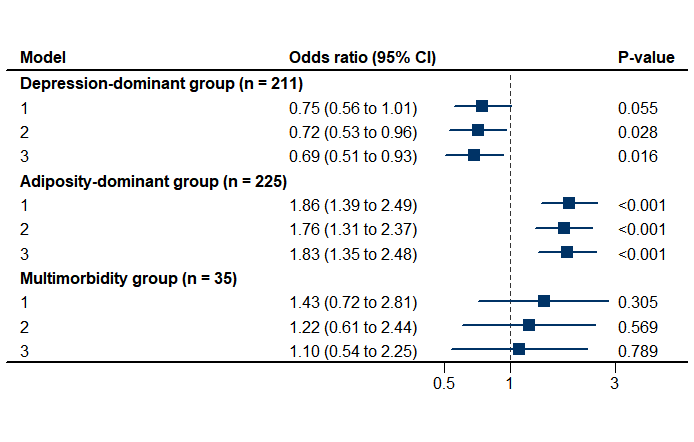


**Figure S4 Sex disparity in the joint trajectories of depression Z-score and BMI Z-score among individuals selected based on the Chinese standards**

**with complete covariate data (before multiple imputations)**

Model 1: multinomial logistic regression model without covariate;

Model 2: multinomial logistic regression model adjusted for individual-level covariates, including age, area, region, high birth weight, premature, and the only child;

Model 3: multinomial logistic regression model adjusted for individual- and family-level covariates, including age, area, region, high birth weight, premature, and the only child, father’s and mother’s education, obese status of father and mother, depression status of father and mother, per capita household net income, left-behind children, and primary caregiver.


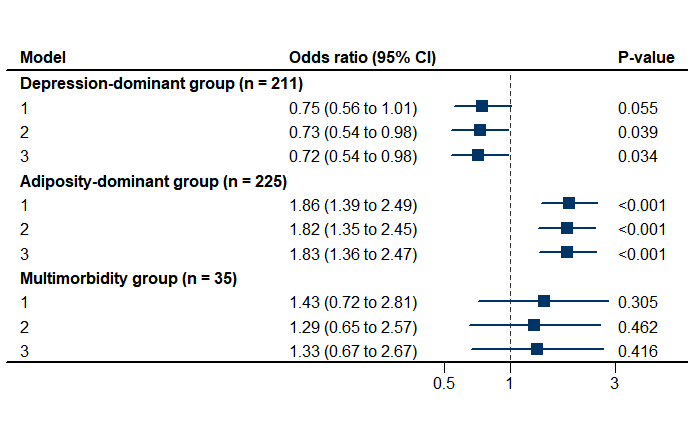


**Figure S5 Sex disparity in the joint trajectories of depression Z-score and BMI Z-score among individuals selected based on the Chinese standards**

**with complete covariate data (after multiple imputations)**

Model 1: multinomial logistic regression model without covariate;

Model 2: multinomial logistic regression model adjusted for individual-level covariates, including age, area, region, high birth weight, premature, and the only child;

Model 3: multinomial logistic regression model adjusted for individual- and family-level covariates, including age, area, region, high birth weight, premature, and the only child, father’s and mother’s education, obese status of father and mother, depression status of father and mother, per capita household net income, left-behind children, and primary caregiver.

**
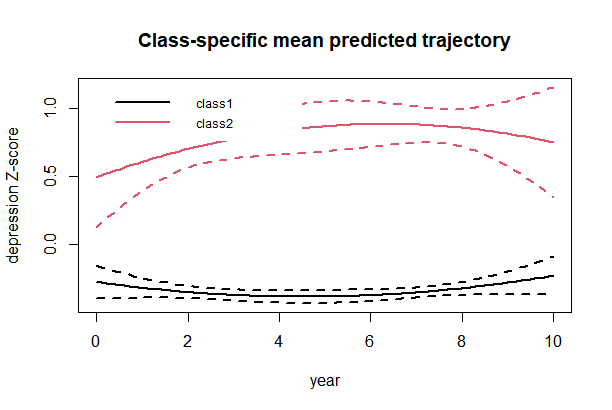
Figure S6 Classification of depression Z-score trajectory**

**among individuals selected based on the WHO standards, excluding the data from 2020 (during the COVID-19 pandemic)**

Class1: 1735 individuals; Class 2: 245 individuals

**
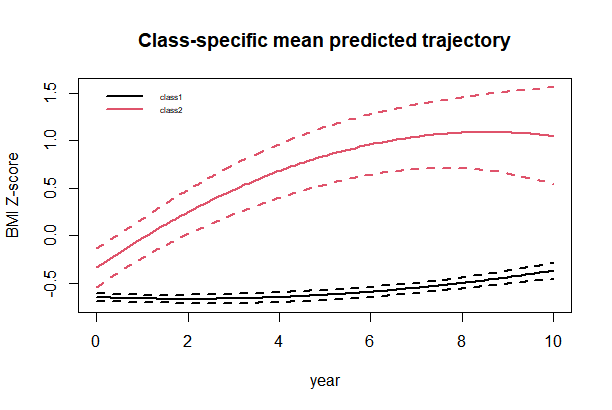
**

**Figure S7 Classification of BMI Z-score trajectory**

**among individuals selected based on the WHO standards, excluding the data from 2020 (during the COVID-19 pandemic)**

Class1: 1867 individuals; Class 2: 113 individuals

**
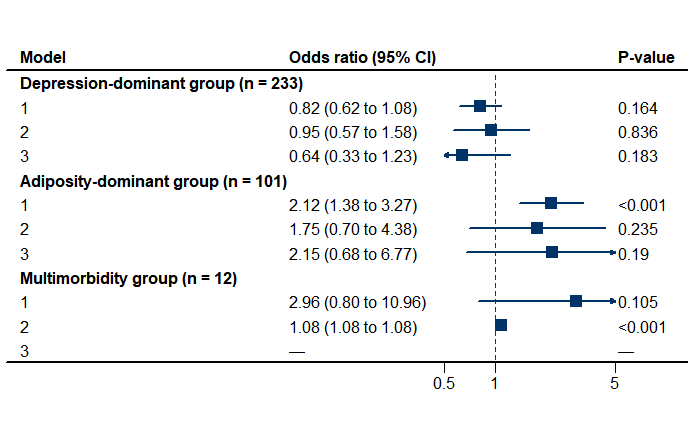
**

**Figure S8 Sex disparity in the joint trajectories of depression Z-score and BMI Z-score among individuals selected based on the WHO standards**

**with complete covariate data, excluding the data from 2020 (before multiple imputations)**

Model 1: multinomial logistic regression model without covariate;

Model 2: multinomial logistic regression model adjusted for individual-level covariates, including age, area, region, high birth weight, premature, and the only child;

Model 3: multinomial logistic regression model adjusted for individual- and family-level covariates, including age, area, region, high birth weight, premature, and the only child, father’s and mother’s education, obese status of father and mother, depression status of father and mother, per capita household net income, left-behind children, and primary caregiver.

1. **Male (B) Female**

**
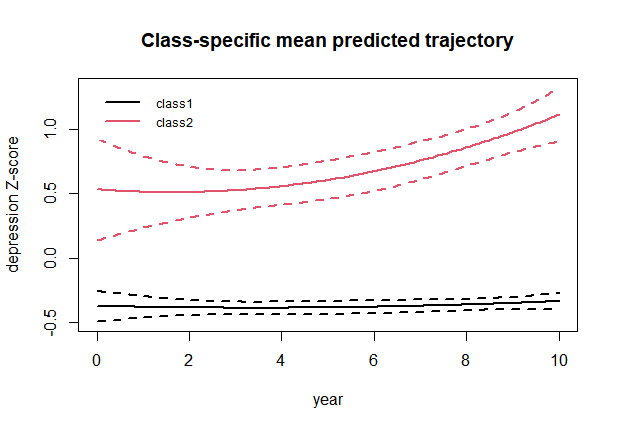

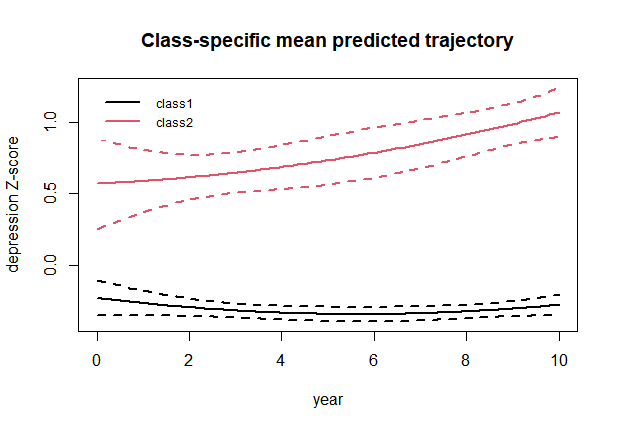
**

**Figure S9 Classification of depression Z-score trajectory**

**among males and females selected based on the WHO standards, respectively**

Males: Class1: 971 individuals; Class 2: 141 individuals; Females: Class1: 897 individuals; Class 2: 159 individuals

1. **Male (B) Female**

**
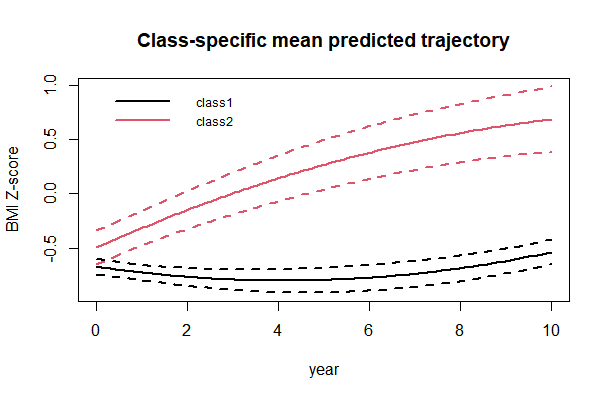

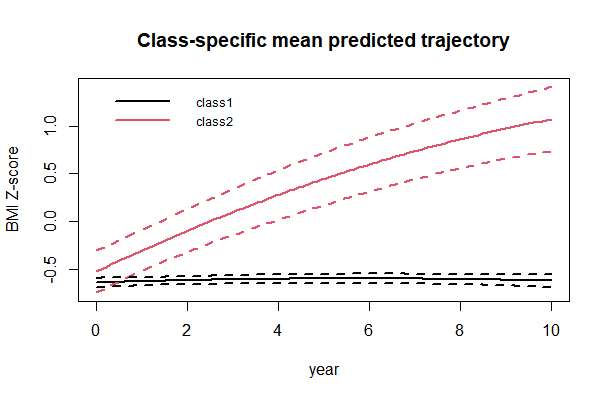
**

**Figure S10 Classification of BMI Z-score trajectory**

**among males and females selected based on the WHO standards, respectively**

Males: Class1: 841 individuals; Class 2: 271 individuals; Females: Class1: 976 individuals; Class 2: 80 individuals
